# Supplementary material for: The Protein Composition Changed the Quality Characteristics of Plant-Based Meat Analogues Produced by a Single-Screw Extruder: Four Main Soybean Varieties in China as Representatives
Source: Foods. 2022 Apr 13;11(8):1112. doi: 10.3390/foods11081112 (PMC9032996; doi:10.3390/foods11081112)
Supplement: Supplementary file 1 [file foods-11-01112-s001.zip › foods-1670832-supplementary.pdf]

Table S1 The amino acid composition of four kinds of plant-based meat analogues (PBMs) (%)

	Heihe 43	Jiyu 86	Suinong 52	Shengfeng 5
Threonine	4.43±0.03a	4.48±0.02a	4.89±0.02b	5.12±0.02c
Valine	5.18±0.01a	5.08±0.08b	5.32±0.09c	5.63±0.09s
Methionine	1.11±0.02a	1.00±0.01b	1.19±0.01c	1.24±0.01d
Isoleucine	4.88±0.01a	4.61±0.03b	4.85±0.01c	4.96±0.01d
Leucine	8.72±0.02a	8.50±0.06b	8.99±0.00c	9.07±0.01d
Phenylalanine	6.79±0.00a	6.40±0.02b	6.54±0.01c	6.80±0.03a
Lysine	7.07±0.01b	7.10±0.02a	6.65±0.01b	6.84±0.01c
Aspartate	11.88±0.02a	11.84±0.02a	11.61±0.02b	11.52±0.01c
Serine	5.93±0.04a	5.74±0.01b	5.92±0.03a	5.66±0.03c
Glutamate	19.71±0.00a	19.54±0.04b	20.08±0.02c	19.45±0.00d
Glycine	4.24±0.01a	4.35±0.02b	3.98±0.02c	4.30±0.00d
Alanine	4.16±0.02a	4.28±0.01b	4.39±0.03c	4.64±0.01d
Tyrosine	2.53±0.01a	2.53±0.10a	2.88±0.05b	2.50±0.03a
Histidine	2.67±0.05a	2.84±0.05b	2.44±0.01c	2.46±0.01c
Arginine	7.16±0.03a	7.31±0.02b	7.36±0.02c	7.35±0.02c
Proline	3.54±0.00a	4.40±0.01b	2.90±0.06c	2.46±0.02d

Different lowercase letters indicate a significant difference ($p < 0.05$)

Table S2 Volatile flavor compounds of the PBM produced by Heihe 43

No.	Relative content (%)	Components	Molecular weight	CAS
1	1.15	dl-Alanine ethyl ester	117.079	017344-99-9
2	0.70	Ethyl 8-p-[[[diethylsulfamyl]phenethyl]amino]-3-methylpyrido[2,3-b]pyrazine-6-carbamate	486.205	021271-98-7
3	1.68	2-Hexanone, 4-methyl-	114.104	000105-42-0
4	1.35	Acetic acid	60.021	000064-19-7
5	2.18	Pentanal	86.073	000110-62-3
6	0.77	Toluene	92.063	000108-88-3
7	11.94	Hexanal	100.089	000066-25-1
8	0.73	2-Heptanone	114.104	000110-43-0
9	1.04	3,3,3-Trifluoro-N-(2-fluorophenyl)-2-(trifluoromethyl)propionamide	289.034	304444-11-9
10	9.13	2-Heptenal, (E)-	112.089	018829-55-5
11	2.53	Benzaldehyde	106.042	000100-52-7
12	1.94	Hexanoic acid	116.084	000142-62-1
13	1.12	N-[5-(Trifluoromethyl)-1,3,4-thiadiazol-2-yl]benzamide	273.018	054047-24-4
14	10.65	1-Octen-3-ol	128.12	003391-86-4
15	1.25	3,4-Dinitrobenzonitrile	193.012	004248-33-3
16	1.50	Furan, 2-pentyl-	138.104	003777-69-3
17	1.24	4-Piperidinecarboxamide	128.095	039546-32-2
18	2.12	1,4-Pentadiene	68.063	000591-93-5
19	1.02	Hexenal, 2-ethyl-	126.104	026266-68-2
20	2.03	2-Tridecenal, (E)-	196.183	007069-41-2
21	2.94	2-Decen-1-ol, (E)-	156.151	018409-18-2
22	1.56	1-Propene, 1-chloro-2-methyl-	90.024	000513-37-1
23	1.25	Furan, 4-methyl-2-propyl-	124.089	006148-37-4
24	0.89	Anthiaergostan-5,7,9,22-tetraen, 15-[2,4-dinitrophenylazo]-	572.336	1000213-78-6
25	1.88	Butane, 2-chloro-	92.039	000078-86-4
26	3.31	Maltol	126.032	000118-71-8
27	1.16	3-Fluoro-4-nitrotoluene	155.038	000446-34-4
28	0.64	2H-Pyran, 2-(2,5-hexadiynyloxy)tetrahydro-	178.099	040924-58-1
29	1.07	Pentanol, 5-amino-	103.1	002508-29-4
30	2.62	Nonadecane	268.313	000629-92-5
31	0.92	5H-Cyclohepta-1,4-dioxin, 2,3,4a,6,7,9a-hexahydro-, cis-	154.099	055956-39-3
32	1.09	4-t-Butyl-2-[3,4-dichlorophenyl]-6-pyrrolodinylmethyl phenol	377.131	1000227-33-2
33	1.74	1-Chloroeicosane	316.29	042217-02-7
34	3.94	2,4-Decadienal, (E,E)-	152.12	025152-84-5
35	0.66	(4Z)-5-Chloro-3,4-dimethyl-2,4-heptadiene	158.086	105949-75-5

36	0.81	tert-Butyl N-(4-nitrophenyl)carbamate	238.095	018437-63-3
37	1.18	Acetamide, 2-chloro-N,N-di-2-propenyl-	173.061	000093-71-0
38	1.25	Cycloheptane, methoxy-	128.12	042604-04-6
39	7.95	4H-Pyrazole, 3-tert-butylsulfanyl-4,4-bistrifluoromethyl-	292.047	1000303-59-8
40	1.06	17-Bromopregnane-3,20-dione	394.151	062973-42-6
41	0.85	2-(4-Chloro-phenoxy)-nicotinic acid	249.019	1000296-88-9
42	0.79	Benzene, 1-azido-4-nitro-	164.033	001516-60-5
43	2.94	Myo-Inositol, 4-C-methyl-	194.079	000472-95-7
44	0.73	Furfurylideniminosulphur pentafluoride	220.993	090598-15-5
45	0.69	Biphenyl, 2,2',4,5',6-pentabromo-	543.631	059080-39-6

Table S3 Volatile flavor compounds of the PBM produced by Jiyu 86

No.	Relative content (%)	Components	Molecular weight	CAS
1	1.34	dl-Alanine ethyl ester	117.079	017344-99-9
2	0.52	Cystine	240.024	000056-89-3
3	0.56	Ethyl 8-p-[[[diethylsulfamyl]phenethyl]amino]-3-methylpyrido[2,3-b]pyrazine-6-carbamate	486.205	021271-98-7
4	1.28	1,3-Propanediamine, N,N,2-trimethyl-N'-(4-nitrophenyl)-	237.148	055667-52-2
5	1.24	Acetic acid	60.021	000064-19-7
6	0.33	2-Butenal	70.042	004170-30-3
7	2.15	Pentanal	86.073	000110-62-3
8	0.74	Butanoic acid, 3-oxo-, 2,2,2-trichloroethyl ester	231.946	058547-15-2
9	0.49	2H-Pyran, 4-chlorotetrahydro-	120.034	001768-64-5
10	0.98	Spiro[2.4]hepta-4,6-diene	92.063	000765-46-8
11	10.69	Hexanal	100.089	000066-25-1
12	0.51	2-Butenediamide, 2-methyl-, (Z)-	128.059	041138-17-4
13	0.50	2H-Pyran, 2-(2,5-hexadiynyloxy)tetrahydro-	178.099	040924-58-1
14	0.43	1-Acrylonitryl-3,3,-dimethyldiaziridine	123.08	077376-92-2
15	0.32	1,3-Cyclopentadiene, 5-(1-methylethylidene)-	106.078	002175-91-9
16	0.73	2H-Pyran, 3,4-dihydro-2-methoxy-	114.068	004454-05-1
17	0.43	1-(Prop-2-ynyl)-3,3-bis(trifluoromethyl)diaziridine	218.028	083391-92-8
18	1.07	1,9-Diaminononane	158.178	000646-24-2
19	14.26	2-Heptenal, (E)-	112.089	018829-55-5
20	4.07	Benzaldehyde	106.042	000100-52-7
21	9.27	1-Nonen-3-ol	142.136	021964-44-3
22	2.33	2-n-Butyl furan	124.089	004466-24-4
23	0.88	Benzene, 1,3,5-trimethyl-	120.094	000108-67-8
24	1.58	Octanal	128.12	000124-13-0
25	0.98	Cyclobutane, (1-methylethylidene)-	96.094	001528-22-9
26	0.40	Chloromethylphosphonic dichloride	165.891	001983-26-2
27	1.85	4-Nonyne	124.125	020184-91-2
28	1.05	3-Hepten-2-one, 3-methyl-	126.104	039899-08-6
29	2.93	1,2,4-Benzothiadiazine, 7-chloro-1, 1-dioxide-4-methyl-3-(4-methyl-1-piperazinyl)-	328.076	070443-35-5
30	3.48	2-Decen-1-ol	156.151	022104-80-9
31	1.11	3,5-Octadien-2-one	124.089	038284-27-4
32	1.28	Heptadecane	240.282	000629-78-7
33	3.22	Nonanal	142.136	000124-19-6

34	3.97	Maltol	126.032	000118-71-8
35	1.84	Benzoic acid, 2,5-bis(trimethylsiloxy)-, trimethylsilyl ester	370.145	003618-20-0
36	1.09	3-Amino-2-cyclohexenone	111.068	005220-49-5
37	0.52	(4Z)-5-Chloro-3,4-dimethyl-2,4-heptadiene	158.086	105949-75-5
38	0.83	Pyridazine, 3,6-dichloro-	147.96	000141-30-0
39	0.42	Tetryl	287.014	000479-45-8
40	2.73	Dodecane	170.203	000112-40-3
41	2.94	Cycloheptane	98.11	000291-64-5
42	1.61	Tridecane	184.219	000629-50-5
43	8.16	2,4-Decadienal	152.12	002363-88-4
44	0.58	1-Benzyl-2,3,3-trimethyldiaziridine	176.131	077671-19-3
45	1.01	Butylated Hydroxytoluene	220.183	000128-37-0
46	0.33	Acetic acid, dichloro-, ethyl ester	155.974	000535-15-9
47	0.45	2-Butanol, 1-[(2-hydroxyethyl)thio]-	150.071	021428-94-4
48	0.53	1-Propanol, 3-(methylthio)-	106.045	000505-10-2

Table S4 Volatile flavor compounds of the PBM produced by Suinong 52

No.	Relative content (%)	Components	Molecular weight	CAS
1	0.62	Glycyl-dl-alanine	146.069	000926-77-2
2	0.81	(S)-(+)-1,2-Propanediol	76.052	004254-15-3
3	3.03	Acetic acid	60.021	000064-19-7
4	1.47	Pentanal	86.073	000110-62-3
5	0.71	But-2-enamide, N,N-diundecyl-3-methyl-	407.413	1000308-24-5
6	1.21	Toluene	92.063	000108-88-3
7	7.49	Hexanal	100.089	000066-25-1
8	0.57	3,3,3-Trifluoro-N-(4-fluorophenyl)-2-(trifluoromethyl)propionamide	289.034	340138-04-7
9	1.10	Carbonic acid, dimethyl ester	90.032	000616-38-6
10	0.57	Cyclopropanecarboxylic acid, 2,3-dichlorophenyl ester	229.99	1000278-66-6
11	0.70	Bis[1-[2-(2-pyridyl)ethyl]-2-methylbenzimidazol-5-yl] sulfone	536.199	1000253-88-6
12	0.63	2-Hexanone, 4-methyl-	114.104	000105-42-0
13	0.98	2-Chloroethyl 1-propynyl sulfoxide	149.991	1000250-50-5
14	0.81	Pentane, 3-ethyl-2-methyl-	114.141	000609-26-7
15	12.59	2-Heptenal, (Z)-	112.089	057266-86-1
16	4.10	Benzaldehyde	106.042	000100-52-7
17	1.26	1,6-Anhydro-.beta.-D-glucopyranose (levoglucosan)	162.053	000498-07-7
18	0.99	5H-Indeno[1,2-b]pyrazin-5-one, 6,7,8-tribromo-9-(ethoxycarbonyl)-N,N'-diethyl-1,2,3,4-tetrahydro-	547.895	1000149-89-3
19	8.48	1-Octen-3-ol	128.12	003391-86-4
20	1.15	2,3-Octanedione	142.099	000585-25-1
21	1.87	2,4-Nonadienal, (E,E)-	138.104	005910-87-2
22	1.07	Cyclopropane,-1-ethenyloxy,-2,2-dichloro	151.98	042039-18-9
23	0.93	Cyclobut-1-enylmethanol	84.058	089182-08-1
24	1.36	1-Ethyl-5-methylcyclopentene	110.11	097797-57-4
25	1.92	Limonene	136.125	000138-86-3
26	0.79	Cyclohexanebutanoic acid, 2-methyl-3-oxo-, methyl ester	212.141	083406-43-3
27	2.66	6-Methoxy-3-(4-methylpiperazine-1-carbonyl)-chromen-2-one	302.127	325471-63-4
28	2.05	2-Hepten-1-ol, (Z)-	114.104	055454-22-3
29	0.94	Cyclohexanone, 2-(2-propenyl)-	138.104	000094-66-6
30	0.95	Bicyclo[2.2.1]heptane, 2-(2-methyl-1-propenyl)-	150.141	061142-27-6
31	1.07	2,6-Dimethyldecane	170.203	013150-81-7
32	2.87	Nonanal	142.136	000124-19-6
33	4.81	Maltol	126.032	000118-71-8
34	0.96	Pyrazole-5-carboxamide, 4-amino-	126.054	1000273-71-8

35	0.65	Sulfurous acid, diethyl ester	138.035	000623-81-4
36	1.81	Z,Z-3,13-Octadecedien-1-ol	266.261	1000131-10-7
37	2.74	Dodecane	170.203	000112-40-3
38	0.64	Pentane, 1-nitro-	117.079	000628-05-7
39	0.77	Propyl 2-ethylbutanoate	158.131	005129-46-4
40	2.22	Oleic Acid	282.256	000112-80-1
41	1.41	Cyclohexane, 1,4-dichloro-, trans-	152.016	016890-91-8
42	1.66	Eicosane	282.329	000112-95-8
43	9.05	2,4-Decadienal	152.12	002363-88-4
44	0.62	Pipridine, 1-chloroacetyl-	161.061	001440-60-4
45	0.56	2,4(1H,3H)-Pyrimidinedione, 6-chloro-5-nitro-	190.973	006630-30-4
46	1.33	Tridecane	184.219	000629-50-5
47	0.69	Propanamide, 2,2-dimethyl-N-(2,4-diiodophenyl)-	428.909	1000266-63-5
48	1.40	4,6-di-tert-Butyl-m-cresol	220.183	000497-39-2
49	0.94	Cyclohexanol, 2-(aminomethyl)-, trans-	129.115	005691-09-8

Table S5 Volatile flavor compounds of the PBM produced by Shengfeng 5

No.	Relative content (%)	Components	Molecular weight	CAS
1	0.59	Urea, N,N'-diethyl-	116.095	000623-76-7
2	0.82	1-Butaneboronic acid	102.085	004426-47-5
3	1.54	1-Propanol, 3-chloro-	94.019	000627-30-5
4	1.64	Acetic acid	60.021	000064-19-7
5	1.64	Benzeneethanamine, 3-benzyloxy-2-fluoro-.beta.-hydroxy-N-methyl-	275.132	103439-01-6
6	0.54	1-Benzyl-4-nitroimidazole	203.069	013230-13-2
7	0.92	Pyridine-3-carboxamide, 1,2-dihydro-4,6-dimethyl-2-thioxo-	182.051	079927-21-2
8	0.60	Toluene	92.063	000108-88-3
9	11.90	Hexanal	100.089	000066-25-1
10	0.73	2,5-Furandione, dihydro-3-methylene-	112.016	002170-03-8
11	0.66	4,5-Thiepanedione, 3,3,6,6-tetramethyl-	200.087	002800-87-5
12	0.88	Methanaminium, 1-carboxy-N,N,N-trimethyl-, hydroxide, inner salt	117.079	000107-43-7
13	1.06	3,4,5,6-Tetrahydro-2,4,6-trimethyl-2H-1,3,5-thiadiazine	146.088	053897-63-5
14	14.00	2-Heptenal, (Z)-	112.089	057266-86-1
15	2.30	Benzaldehyde	106.042	000100-52-7
16	0.78	Heptanoic acid	130.099	000111-14-8
17	8.07	1-Octen-3-ol	128.12	003391-86-4
18	1.63	Furan, 2-pentyl-	138.104	003777-69-3
19	0.76	Benzene, 1,2,4-trimethyl-	120.094	000095-63-6
20	0.63	2-(Chloromethyl)-5-oxo-1,2-oxaphospholane 2-oxide	167.974	022793-96-0
21	1.77	4-(4-Methylaminobutyl)-5-(4-nitrophenyl)-2-(2-piperidino-1-(4-chlorpheny)vinyl-thiazole	510.186	155243-64-4
22	1.16	3,5-Octadien-2-ol	126.104	069668-82-2
23	3.25	5-Ethyl-1-nonene	154.172	019780-74-6
24	1.03	Cyclohexanone, 2-(1-mercapto-1-methylethyl)-5-methyl-	186.108	038462-22-5
25	1.22	Furan, 4-methyl-2-propyl-	124.089	006148-37-4
26	1.48	Nonadecane	268.313	000629-92-5
27	3.51	2-Decen-1-ol, (E)-	156.151	018409-18-2
28	1.12	Maltol	126.032	000118-71-8
29	0.52	1,8-Nonadien-3-ol	140.12	159010-02-3
30	1.46	1-Dodecene	168.188	000112-41-4
31	0.72	Benzofurazan, 5-nitro-	165.017	018772-11-7
32	0.63	Phenol, 2-chloro-	128.003	000095-57-8
33	4.19	Dodecane	170.203	000112-40-3
34	0.88	1,4-Cyclohexanedimethanamine	142.147	002549-93-1
35	4.44	2-Methylene cyclopentanol	98.073	020461-31-8

36	1.48	2,5,9-Tetradecatriene, 3,12-diethyl-	248.25	074685-87-3
37	3.44	Hexadecane	226.266	000544-76-3
38	7.17	2,4-Decadienal	152.12	002363-88-4
39	0.90	1-Propene-1,2,3-tricarboxylic acid	174.016	000499-12-7
40	0.53	1,3-Bis(2-chloroethyl)urea	184.017	002214-72-4
41	1.01	Tetradecane	198.235	000629-59-4
42	1.05	2H-Pyran-5-carboxamide, 2-oxo-N-(3-chlorophenyl)-	249.019	339241-73-5
43	0.93	Procyazine	252.089	032889-48-8
44	3.08	Butylated Hydroxytoluene	220.183	000128-37-0
45	0.52	2H-Pyran, 2-(2,5-hexadiynyloxy)tetrahydro-	178.099	040924-58-1
46	0.80	Propanenitrile, 3-chloro-	89.003	000542-76-7
